# The gut microbiota dysbiosis in ischemic stroke: a systematic review and meta-analysis of microbial diversity and taxonomic alterations

**DOI:** 10.3389/fnagi.2026.1914614

**Published:** 2026-08-31

**Authors:** Heng Yang, Xu Li, Zhen Cao, Xiaofeng Zeng, Yan He, Jiagui Huang

**Affiliations:** 1 Department of Neurology, Second People’s Hospital of Yibin City-West China Yibin Hospital, Sichuan University, Yibin, China; 2 Department of Geriatrics, Second People’s Hospital of Yibin City-West China Yibin Hospital, Sichuan University, Yibin, China

**Keywords:** diversity, gut microbiota, ischemic stroke, meta-analysis, systematic review, taxonomy

## Abstract

**Objective:**

Clinical and epidemiological evidence suggests a link between gut microbiota and ischemic stroke severity as well as outcomes, yet inconsistent findings and unclear mechanisms underscore the need for a systematic synthesis.

**Methods:**

We systematically reviewed and meta-analyzed observational studies linking gut microbiota to ischemic stroke, following PRISMA guidelines. The search covered PubMed, Embase, and the Cochrane Library, with animal studies and *in vitro* studies excluded. Two reviewers independently screened the literature, extracted data, and assessed study quality via the Newcastle–Ottawa Scale. We conducted random-effects meta-analyses using RevMan 5.3. Heterogeneity was quantified with the *I*^2^ statistic, and publication bias was assessed. Subgroup analyses were conducted to examine influential factors.

**Results:**

From 17,281 initially identified records, 59 studies involving 7,463 patients were included in the final analysis. Although α-diversity measures—including Shannon, Chao1, ACE, Simpson index, and observed species richness—did not differ significantly between ischemic stroke patients and controls (all *p* > 0.05), subgroup analyses indicated that observed species richness (95% CI [−0.80, −0.13], *p* = 0.006) was notably altered in patients with cognitive impairment, and the Chao1 index (95% CI [0.01, 0.37], *p* = 0.04) was significantly changed in those with mood disorders. Microbial composition analysis revealed no significant differences at the phylum level. However, at the family level, stroke patients exhibited a higher abundance of *Enterobacteriaceae* and lower levels of *Lachnospiraceae* and *Prevotellaceae*. At the genus level, these patients exhibited higher levels of *Pseudomonas* and *Streptococcus*, along with reduced abundances of *Akkermansia*, *Bacteroides*, *Coprococcus*, and *Roseburia*.

**Conclusion:**

Ischemic stroke is characterized by taxonomic gut dysbiosis, whereas a decrease in α-diversity is observed only in patients who also develop cognitive impairment and mood disorders after stroke. Future multi-omics and large cohort studies should establish causality between gut microbiota and ischemic stroke, identifying therapeutic targets.

**Systematic review registration:**

https://www.crd.york.ac.uk/PROSPERO/view/CRD42024506656, Identifier: CRD42024506656.

## 1 Introduction

Ischemic stroke leads to significant disability and mortality across the globe, posing a major threat to public health. Given the short treatment time windows and limited availability of specialized centers, most patients are unable to receive timely intravenous thrombolysis or endovascular thrombectomy. Moreover, despite their ability to reestablish cerebral blood flow, these interventions do not reduce downstream tissue injury or improve functional recovery. Although various neurorestorative strategies are being actively explored to facilitate post-stroke recovery, their clinical application is still limited by an incomplete understanding of the underlying mechanisms. This knowledge gap highlights the pressing need for novel therapeutic approaches that can lessen the stroke burden and improve patient outcomes.

Mounting evidence now links the gut microbiota to brain disorders (Hou et al., 2022). Aging induces profound restructuring of the gut microbial ecosystem, characterized by a loss of microbial diversity, a decline in beneficial bacterial populations, and an increase in opportunistic pathogens (Bárcena et al., 2019). Accumulating research indicates that the gut microbiota is critically involved in ischemic stroke through the gut–brain axis, modulating neuroinflammatory responses, behavioral performance, and cognitive function (Peh et al., 2022; Pilon et al., 2025). Meanwhile poor stroke recovery is associated with characteristic gut dysbiosis (Lledós et al., 2025). Stroke-induced damage to the intestinal barrier may allow gut microbes to enter systemic circulation, potentially worsening inflammation (Stanley et al., 2016). Gut dysbiosis is further linked to worsened stroke outcomes, such as higher risks of pneumonia, malnutrition, and mortality (Zhang et al., 2024). An early meta-analysis linked elevated α-diversity to post-stroke depression (Li et al., 2025), whereas cognitive impairment after stroke was associated with reduced Simpson diversity (Hu et al., 2023).

While prior reviews have touched on the gut microbiota–stroke axis, the scattered and often inconsistent findings underscore the need for a rigorous systematic synthesis. Therefore, this meta-analysis aims to synthesize clinical evidence on gut microbiota alterations in ischemic stroke, with subgroup analyses focusing on post-stroke cognitive impairment, mood disorders, and infection.

## 2 Methods

This review’s protocol was registered prospectively with PROSPERO (CRD42024506656). Our methods followed the PRISMA (Preferred Reporting Items for Systematic Reviews and Meta-Analyses) standards and Cochrane guidance for overviews and updates of reviews.

### 2.1 Literature retrieval and data sources

A systematic search was conducted across PubMed, the Cochrane Library, and Embase via Ovid SP in October 2025 using terms related to (“ischemic stroke,” “acute ischemic stroke,” “stroke recovery,” “stroke unit,” “acute cerebral infarction,” “cerebral infarction,” “cerebral ischemia,” and “infarction”) combined with terms associated with gut microbiota (including “gut microbiota,” “gut microbiome,” “dysbiosis,” “gut dysbiosis,” “microbiota,” “probiotics,” “gut-brain axis,” and “microbiota-gut-brain axis”). Supplementary Table S1 provides a detailed description of the search strategy. A manual search was performed on the cited references and relevant review papers to uncover other studies that met our inclusion criteria.

### 2.2 Selection criteria

Two reviewers (HY and XL) independently screened titles and abstracts, with full texts retrieved when either reviewer deemed them potentially relevant. To evaluate inter-reviewer reliability, Cohen’s *κ* was computed on a random 10% sample of the records. The resulting *κ* value was 0.87 (95% CI: 0.79–0.93), indicating almost perfect agreement. All disputes were settled through dialogue, or when necessary, arbitration by an independent third reviewer (YH).

Eligible studies were: (a) observational research (cross-sectional, case–control, or cohort) investigating the relationship between gut microbiota and ischemic stroke; (b) study populations with a mean or median age of 18 years or older; (c) clearly defined diagnostic criteria for ischemic stroke; (d) standardized assessment of gut microbiota; (e) publications in English; and (f) data presented as mean ± standard deviation (SD). We excluded studies that used *in vitro* or animal experiments. For cohorts that overlapped across multiple studies, we kept only the one with the largest sample size. In studies that reported multiple patient groups, comparisons were performed between healthy controls and the most severe stroke subgroup, and baseline data from eligible interventional studies were also integrated.

### 2.3 Data extraction

XL and ZC each independently performed the task of extracting data from the included studies. The dataset included key study characteristics (such as publication year, first author, and cohort), study design, cohort size, proportion of female participants, gut microbiota outcomes, and the methods employed to evaluate both ischemic stroke and gut microbiota. We used Web Plot Digitizer to extract data from figures when raw data were not directly available. For prospective studies, baseline characteristics were collected. A third reviewer (YH) supervised the extraction process, and any discrepancies were resolved through group discussion. When multiple adjusted models were available, we selected the one that controlled for the most relevant confounders.

### 2.4 Quality assessment

Using the Newcastle–Ottawa Scale (NOS), JH and XZ independently appraised the methodological quality of population-based studies (Margulis et al., 2014). A validated nine-item tool for observational studies that rates selection, comparability, and outcome assessment from 0 to 9. Scores were divided into three quality tiers: high (≥7), moderate (4–6), and low (<4).

### 2.5 Statistical analysis

Statistical analyses were conducted in RevMan 5.3 (Cochrane Collaboration, http://ims.cochrane.org/revman) and STATA/SE 15.1 (StataCorp, College Station, TX, USA using the metaninf package). Statistical analyses were performed using RevMan 5.3 (Cochrane Collaboration, Oxford, UK) for the primary meta-analyses, and Stata/SE 15.1 (StataCorp LLC, College Station, TX, USA) for all supplementary analyses. These included sensitivity analysis (leave-one-out influence diagnostics via the metan command with the inf option), meta-regression, and publication bias assessment (Egger’s and Begg’s tests). All continuous outcomes, including α-diversity indices and microbial relative abundances, were combined using the standardized mean difference (SMD) with Hedges’g correction to account for differences in measurement scales and methodologies across studies. A random-effects model (DerSimonian–Laird) was used to estimate overall effect sizes, given anticipated clinical and methodological heterogeneity. A two-sided *p*-value below 0.05 served as the criterion for statistical significance. This model incorporates both within- and between-study variability. To assess heterogeneity, we applied the Cochrane Q test together with the *I*^2^ statistic, with *I*^2^ > 50% representing substantial heterogeneity. Quantitative synthesis was conducted only for outcomes that appeared in at least two studies; results from single studies were reported narratively. To examine the robustness of the results, sensitivity analyses were performed to assess the effect of removing studies that had inconsistent results or low methodological quality. To detect publication bias, we applied Begg’s and Egger’s tests, as well as further evaluating its potential impact with the trim-and-fill method.

## 3 Results

### 3.1 Literature search

**Figure 1:**
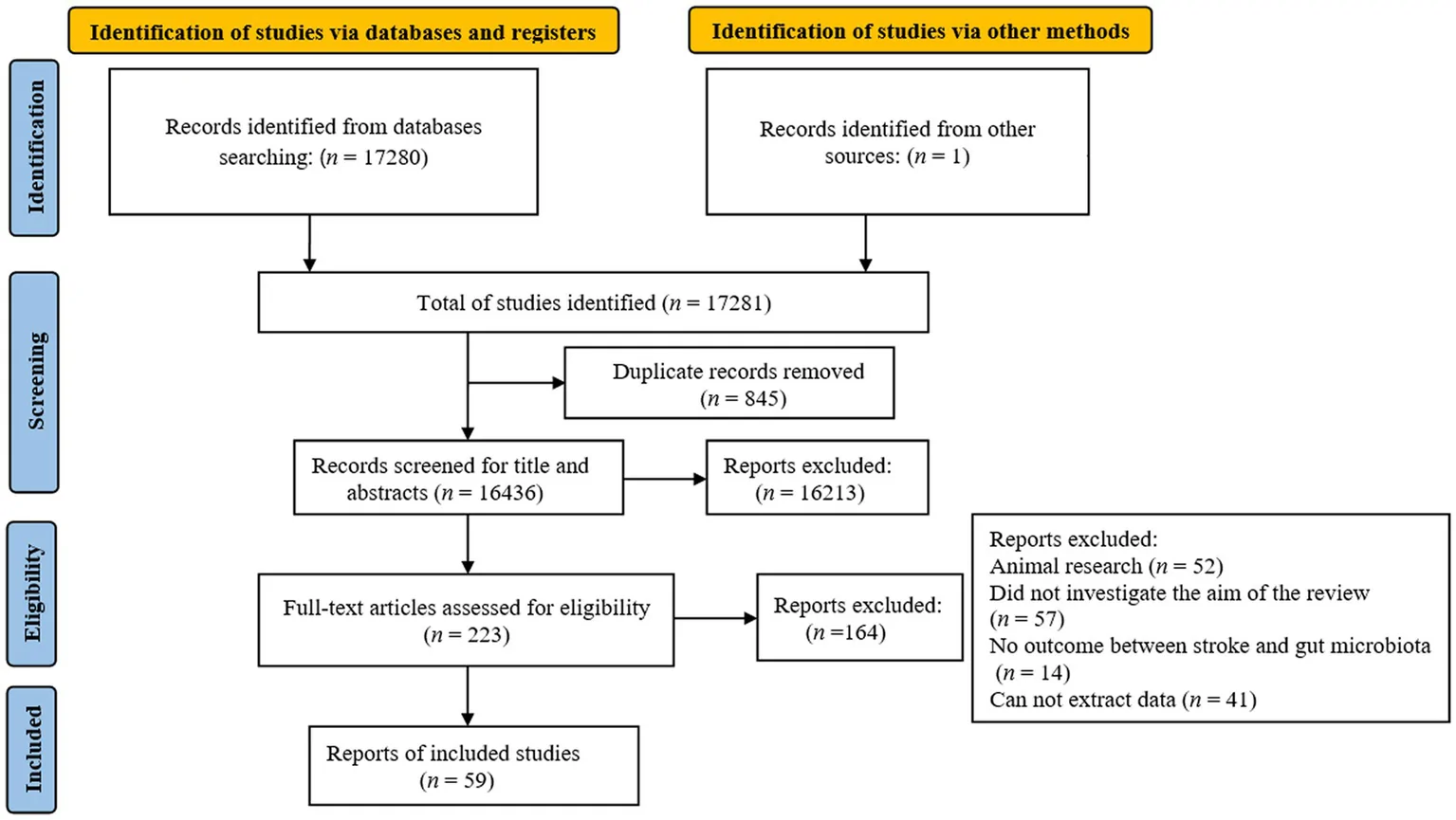
PRISMA flow chart for selection of papers for systematic review.

As depicted in Figure 1, the PRISMA flowchart illustrates the study selection process for this review. 17,281 records were found at the initial stage. After eliminating 845 duplicates, we reviewed the titles and abstracts and excluded 16,213 studies. The remaining 223 articles underwent a thorough full-text assessment, and 59 of them were finally included in the meta-analysis (Bao et al., 2024; Cao N. et al., 2025; Cao Y. et al., 2025; Chang et al., 2021; Chen et al., 2021; Chen et al., 2022; Chen et al., 2024; Chen et al., 2025; Chou et al., 2023; Cui et al., 2023; Dang et al., 2021; Fan et al., 2016; Fang et al., 2025; Feng et al., 2021; Gu et al., 2021; Guo et al., 2021; Haak et al., 2021; He et al., 2024; Huang et al., 2021; Huang et al., 2024; Huang et al., 2025; Jeng et al., 2025; Jiang Y. et al., 2023; Jiang Z. et al., 2023; Jiang et al., 2024; Kang et al., 2021; Li et al., 2019; Li et al., 2020; Li T. et al., 2022; Li Y. M. et al., 2022; Li et al., 2023; Liang et al., 2025; Lin et al., 2025; Ling et al., 2020a; Ling et al., 2020b; Liu et al., 2020; Lledós et al., 2025; Lou et al., 2024; Luo et al., 2024; Ma et al., 2024; Pilon et al., 2025; Shi et al., 2023; Sun et al., 2022; Tan et al., 2021; Wang et al., 2018; Wei et al., 2023; Wei et al., 2024; Wen et al., 2025; Wu et al., 2025; Xia et al., 2021; Xiang et al., 2020; Xu et al., 2021; Yao et al., 2023; Yin et al., 2015; Yoshimura et al., 2025; Yu et al., 2024; Zhang et al., 2023; Zhao et al., 2022; Zheng et al., 2022).

### 3.2 Study characteristics

Fifty-nine studies involving 7,463 patients were comprised in this analysis, consisting of 37 retrospective analyses and 22 prospective studies, 11 of which were cohort studies. The included studies were conducted in five countries, with the majority originating from China. The NOS was employed to evaluate the quality of the methods in the population-based studies. Table 1 and Supplementary Table S2 summarize the baseline characteristics of the included studies.

**Table 1:** Characteristics of each study included in the meta-analysis.

Study (First Author, Year)	Sample sizes	Female (%)	Mean age (Years)^a^	Country	Study design	Intestinal microbiota detection	Nos
Wei et al. (2024)	332	123 (37.05)	66.93	China	Cross-sectional study	16 s rRNA V3-V4	5/9
Jiang et al. (2024)	100	49 (49.00)	56.93	China	Cross-sectional study	16 s rRNA V3-V4	5/9
Lou et al. (2024)	48	20 (41.67)	63.69	China	Cross-sectional study	16S rRNA V4	5/9
Sun et al. (2022)	132	43 (32.58)	64.50	China	Prospective observational cohort study	16S rRNA V3-V4	8/9
Lin et al. (2025)	80	34 (42.50)	72.50	China	prospective study	16S rRNA	9/9
Gu et al. (2021)	135	44 (32.59)	67.98	China	Prospective observational cohort study	16S rRNA V3-V4	7/9
Chen et al. (2022)	231	79 (34.19)	65.76	China	Prospective cohort study	16S rRNA	7/9
Guo et al. (2021)	79	29 (36.71)	—	China	Prospective observational study	16S rRNA V3-V4	5/9
Xiang et al. (2020)	46	25 (54.35)	—	China	Cross-sectional study	16S rDNA V3	5/9
Huang et al. (2024)	48	18 (37.50)	60.81	China	Cross-sectional study	16S rRNA V3-V4	5/9
Lledós et al. (2025)	128	51 (39.84)	76.00	Spain	Prospective observational study	16S rRNA	9/9
Yao et al. (2023)	232	84 (36.21)	64.90	China	Prospective study	16 s rRNA V3-V4	7/9
Yu et al. (2024)	338	176 (52.07)	65.00	China	Cross-sectional study	16 s rRNA	4/9
Ma et al. (2024)	130	47 (36.15)	61.53	China	Cross-sectional study	16S rRNA	5/9
He et al. (2024)	90	31 (34.44)	59.33	China	Prospective study	16S rRNA	7/9
Li et al. (2023)	135	44 (32.59)	67.96	China	Prospective study	16S rRNA V3-V4	8/9
Li T. et al. (2022)	27	13 (48.15)	67.04	China	Cross-sectional study	16S rRNA V3-V4	5/9
Luo et al. (2024)	90	31 (34.44)	67.48	China	Cross-sectional study	16S rRNA V3-V4	5/9
Liang et al., 2025	160	42 (26.25)	62.00	China	Prospective observational single-center cohort study	16S rRNA V3-V4	7/9
Bao et al. (2024)	62	24 (38.71)	62.36	China	Cross-sectional study	16S rRNA V3-V4	6/9
Chang et al. (2021)	398	164 (41.21)	63.60	Koreans	Case–control study	16S rRNA V3-V4	6/9
Cao N. et al. (2025)	60	—	—	China	Cross-sectional study	16S rRNA V4	5/9
Cao Y. et al. (2025)	40	17 (42.50)	59.73	China	Cross-sectional study	16S rRNA V3-V4	5/9
Chen et al. (2024)	70	—	59.14	China	Cross-sectional study	16S rRNA V4-V5	6/9
Chen et al. (2025)	40	—	63.60	China	Cross-sectional study	16S rRNA V3-V4	6/9
Chou et al. (2023)	51	22 (43.14)	70.60	China Taiwan	Prospective cohort study	16S rRNA V3-V4	7/9
Cui et al. (2023)	31	12 (38.71)	61.19	China	Prospective cohort study	16S rRNA	5/9
Dang et al. (2021)	73	25 (34.25)	59.27	China	Prospective cohort study	16S rRNA V3-V4	5/9
Pilon et al. (2025)	65	28 (43.08)	67.85	Brazil	cross-sectional observational study	16S rRNA V3-V4	5/9
Fan et al. (2016)	60	—	—	China	Cross-sectional study	16S rRNA V3	2/9
Fang et al. (2025)	134	41 (30.60)	—	China	Cross-sectional study	16S rRNA V3-V4	3/9
Huang et al. (2021)	95	—	—	China	Cross-sectional study	16S rRNA V3-V4	4/9
Huang et al. (2025)	60	13 (21.67)	66.92	China	Cross-sectional study	16S rRNA V3-V4	5/9
Jeng et al. (2025)	95	30 (31.58)	60.50	China Taiwan	Cross-sectional study	16S rRNA V3-V4	6/9
Jiang Y. et al. (2023); Jiang Z. et al. (2023)	150	49 (32.67)	60.36	China	Prospective observational study	16S rRNA V3-V4	7/9
Jiang et al. (2024)	14	6 (42.86)	67.72	China	Cross-sectional study	16S rRNA V4	6/9
Li et al. (2019)	60	21 (35.00)	62.32	China	Case–control study	16S rRNA V1-V2	7/9
Li et al. (2020)	177	70 (39.55)	65.17	China	Case–control study	16S rRNA	7/9
Li et al. (2022b)	36	16 (44.44)	60.30	China	Cross-sectional study	16S rRNA	6/9
Ling et al. (2020a); Ling et al. (2020b)	66	35 (53.03)	—	China	Cross-sectional study	16S rRNA V3-V4	6/9
Liu et al. (2020)	83	16 (19.28)	—	China	Prospective longitudinal cohort study	16 s rRNA V3-V4	7/9
Shi et al. (2023)	65	32 (49.23)	64.80	China	Prospective Cohort Study	16S rRNA V3-V4	5/9
Haak et al. (2021)	400	177 (44.25)	—	The Netherlands	Prospective Case–Control Study	16S rRNA V3-V4	7/9
Chen et al. (2021)	240	—	—	China	Cross-sectional study	16S rRNA V4	2/9
Kang et al. (2021)	163	78 (47.85)	55.91	China	Cross-sectional study	—	2/9
Ling et al. (2020a); Ling et al. (2020b)	135	-	—	China	Prospective Cohort Study	16S rRNA V3-V4	5/9
Tan et al. (2021)	232	86 (37.07)	—	China	Prospective observational study	16S rRNA V4	8/9
Xia et al. (2021)	332	98 (29.52)	—	China	Prospective observational study	16S rRNA V4	8/9
Xu et al. (2021)	132	62 (46.97)	—	China	Case–control study	16S rRNA V4-V5	5/9
Wang et al. (2018)	20	10 (50.00)	64.00	China	Cross-sectional study	16S rRNA V4	3/9
Wei et al. (2023)	66	33 (50.00)	—	China	Cross-sectional study	16S rRNA V3-V4	3/9
Wen et al. (2025)	103	63 (61.17)	—	China	Prospective Cohort Study	16S rRNA	7/9
Wu et al. (2025)	66	34 (51.52)	—	China	Case–control Study	16S rRNA	7/9
Yoshimura et al. (2025)	156	69 (44.23)	78.40	Japan	Cross-sectional study	16S rRNA V3-V4	6/9
Yin et al. (2015)	553	203 (36.71)	—	China	Case–control Study	16S rRNA V4	6/9
Zhang et al. (2023)	40	23(57.50)	61.75	China	Prospective observational case–control	16S rRNA V3-V4	5/9
Zhao et al. (2022)	60	—	—	China	Cross-sectional study	Metagenomic sequencing	4/9
Zheng et al. (2022)	63	26 (41.27)	41.13	China	Cross-sectional study	16S rRNA V3-V4	5/9
Feng et al. (2021)	47	21 (44.68)	60.70	China	Cross-sectional study	16S rRNA	5/9

NOS, Newcastle–Ottawa Scale; rRNA, ribosomal ribonucleic acid.

a Mean or median as reported.

### 3.3 Gut microbiome diversity in ischemic stroke

#### 3.3.1 Association between Shannon index and ischemic stroke

48 studies (Bao et al., 2024; Cao N. et al., 2025; Cao Y. et al., 2025; Chen et al., 2022; Chen et al., 2024; Chen et al., 2025; Chou et al., 2023; Cui et al., 2023; Dang et al., 2021; Fan et al., 2016; Fang et al., 2025; Feng et al., 2021; Gu et al., 2021; Guo et al., 2021; Haak et al., 2021; He et al., 2024; Huang et al., 2021; Huang et al., 2024; Huang et al., 2025; Jeng et al., 2025; Jiang Y. et al., 2023; Jiang Z. et al., 2023; Jiang et al., 2024; Li et al., 2019; Li et al., 2020; Li T. et al., 2022; Li Y. M. et al., 2022; Li et al., 2023; Liang et al., 2025; Ling et al., 2020a; Ling et al., 2020b; Liu et al., 2020; Lou et al., 2024; Luo et al., 2024; Ma et al., 2024; Pilon et al., 2025; Shi et al., 2023; Sun et al., 2022; Wei et al., 2023; Wei et al., 2024; Wen et al., 2025; Wu et al., 2025; Xiang et al., 2020; Yao et al., 2023; Yin et al., 2015; Yoshimura et al., 2025; Yu et al., 2024; Zhang et al., 2023; Zhao et al., 2022; Zheng et al., 2022) analyzed the Shannon index in ischemic stroke, comprised a total of 4,545 patients with a 95% CI of 0.10 [−0.09, 0.30], *p* = 0.30, and *I*^2^ = 89% (Supplementary Figure S1). Subgroup analyses of the Shannon index, based on the 16S rRNA sequencing target region, study quality scores, and geographic region (Asia vs. non-Asia), revealed no statistically significant differences across the subgroups (Supplementary Figures S2–S4).

#### 3.3.2 Association between Chao1 index and ischemic stroke

38 studies (Bao et al., 2024; Cao N. et al., 2025; Cao Y. et al., 2025; Chen et al., 2024; Chou et al., 2023; Cui et al., 2023; Fan et al., 2016; Fang et al., 2025; Feng et al., 2021; Gu et al., 2021; Guo et al., 2021; He et al., 2024; Huang et al., 2021; Huang et al., 2025; Jeng et al., 2025; Jiang Y. et al., 2023; Jiang Z. et al., 2023; Jiang et al., 2024; Li et al., 2019; Li et al., 2020; Li T. et al., 2022; Li Y. M. et al., 2022; Li et al., 2023; Liang et al., 2025; Lin et al., 2025; Liu et al., 2020; Lledós et al., 2025; Lou et al., 2024; Luo et al., 2024; Sun et al., 2022; Wang et al., 2018; Wen et al., 2025; Wu et al., 2025; Xiang et al., 2020; Yao et al., 2023; Yin et al., 2015; Zhang et al., 2023; Zhao et al., 2022; Zheng et al., 2022) analyzed the Chao1 index in ischemic stroke, comprised a total of 2,946 patients with a 95% CI of 0.07 [−0.05, 0.20], *p* = 0.24, and *I*^2^ = 59% (Supplementary Figure S5).

#### 3.3.3 Association between ACE indexes and ischemic stroke

29 studies (Cao N. et al., 2025; Cao Y. et al., 2025; Chen et al., 2022; Chen et al., 2024; Cui et al., 2023; Dang et al., 2021; Fan et al., 2016; Fang et al., 2025; Feng et al., 2021; Gu et al., 2021; Huang et al., 2021; Huang et al., 2025; Jiang et al., 2024; Li et al., 2019; Li et al., 2020; Li T. et al., 2022; Li Y. M. et al., 2022; Li et al., 2023; Lledós et al., 2025; Lou et al., 2024; Shi et al., 2023; Sun et al., 2022; Wei et al., 2023; Wei et al., 2024; Wen et al., 2025; Xiang et al., 2020; Zhang et al., 2023; Zhao et al., 2022; Zheng et al., 2022) analyzed ACE indexes in ischemic stroke, comprised a total of 2,387 patients with a 95% CI of 0.14 [−0.02, 0.30], *p* = 0.08, and *I*^2^ = 67% (Supplementary Figure S6).

#### 3.3.4 Association between Simpson index and ischemic stroke

33 studies (Bao et al., 2024; Cao N. et al., 2025; Cao Y. et al., 2025; Chen et al., 2024; Cui et al., 2023; Fan et al., 2016; Fang et al., 2025; Feng et al., 2021; Gu et al., 2021; Haak et al., 2021; He et al., 2024; Huang et al., 2021; Huang et al., 2024; Huang et al., 2025; Jeng et al., 2025; Jiang Y. et al., 2023; Jiang Z. et al., 2023; Jiang et al., 2024; Li et al., 2019; Li et al., 2020; Li T. et al., 2022; Li Y. M. et al., 2022; Li et al., 2023; Ling et al., 2020a; Ling et al., 2020b; Liu et al., 2020; Lou et al., 2024; Luo et al., 2024; Ma et al., 2024; Pilon et al., 2025; Sun et al., 2022; Wen et al., 2025; Xiang et al., 2020; Yu et al., 2024; Zhang et al., 2023) analyzed the Simpson index in ischemic stroke, comprised a total of 2,733 patients with a 95% CI of −0.13 [−0.34, 0.07], *p* = 0.21, and *I*^2^ = 83% (Supplementary Figure S7). Subgroup analyses stratified by the 16S rRNA sequencing target region, study quality scores, and geographic region (Asia vs. non-Asia) did not reveal any statistically significant differences in the Simpson index across the subgroups (Supplementary Figures S8–S10).

#### 3.3.5 Association between observed species richness and ischemic stroke

15 studies (Bao et al., 2024; Gu et al., 2021; Haak et al., 2021; Jeng et al., 2025; Jiang et al., 2024; Li et al., 2023; Lin et al., 2025; Liu et al., 2020; Lledós et al., 2025; Luo et al., 2024; Sun et al., 2022; Xiang et al., 2020; Yin et al., 2015; Zhao et al., 2022; Zheng et al., 2022) analyzed the observed species richness in ischemic stroke, comprised a total of 1,701 patients with a 95% CI of −0.10 [−0.29, 0.10], *p* = 0.34, and *I*^2^ = 68% (Supplementary Figure S11).

### 3.4 Stratified analysis of microbial α-diversity in ischemic stroke

#### 3.4.1 Gut microbiome α-diversity in post-stroke cognitive impairment

**Figure 2:**
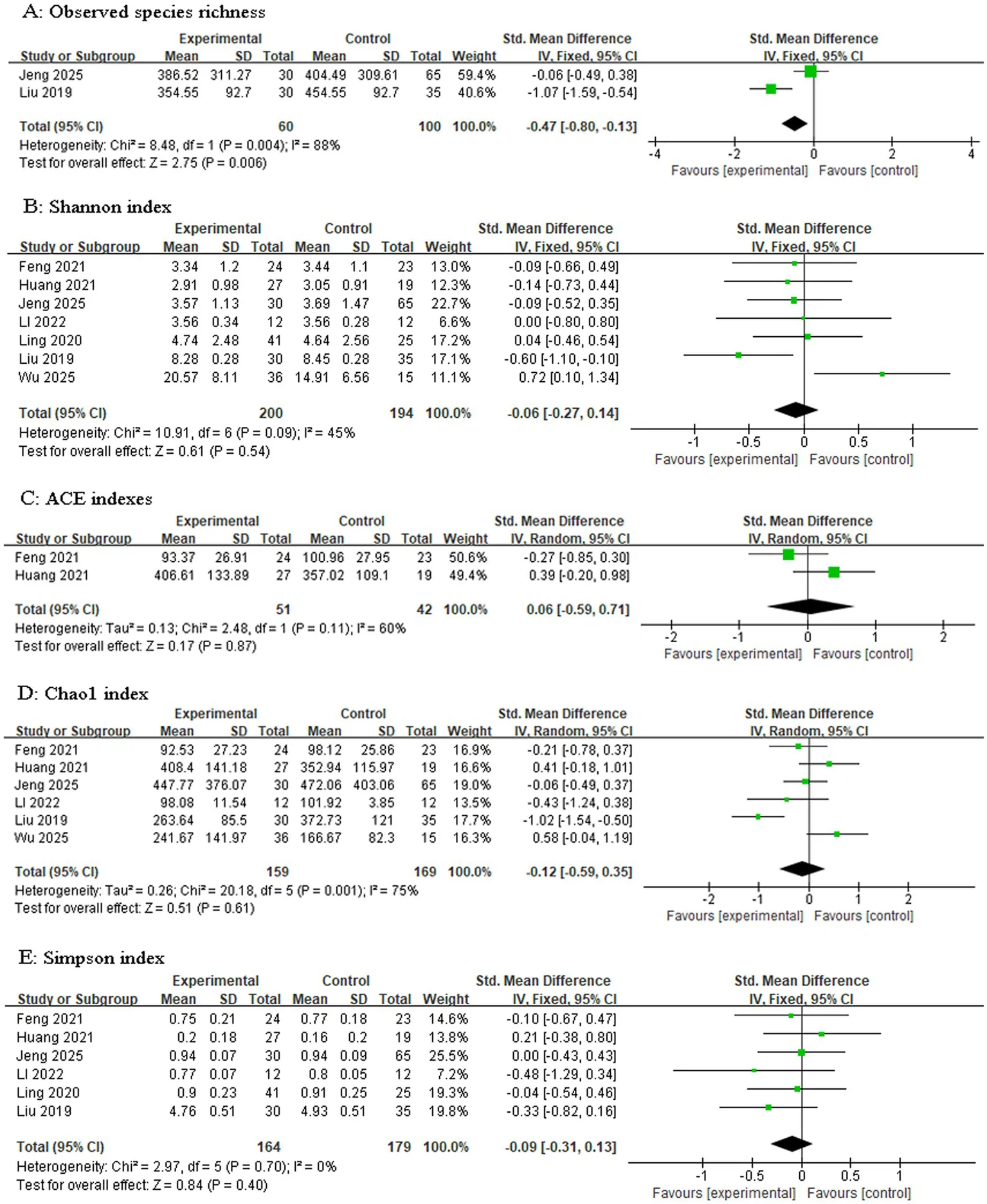
Forest plots comparing the observed species richness **(A)**, Shannon index **(B)**, ACE indexes **(C)**, Chao1 index **(D)**, and Simpson index **(E)** between patients with post-stroke cognitive impairment and healthy controls.

Seven studies (Feng et al., 2021; Huang et al., 2021; Jeng et al., 2025; Li T. et al., 2022; Li Y. M. et al., 2022; Ling et al., 2020a; Ling et al., 2020b; Liu et al., 2020; Wu et al., 2025) investigated the impact of gut microbiota on post-stroke cognitive impairment. The observed species richness showed statistically significant differences between groups (95% CI: −0.47 [−0.80, −0.13], *p* = 0.006, *I*^2^ = 88%) (Figure 2A). However, the remaining α-diversity measures—Shannon index, ACE, Chao1, and Simpson index—did not differ significantly among groups (Figures 2B–E).

#### 3.4.2 Gut microbiome α-diversity in post-stroke mood disorders

**Figure 3:**
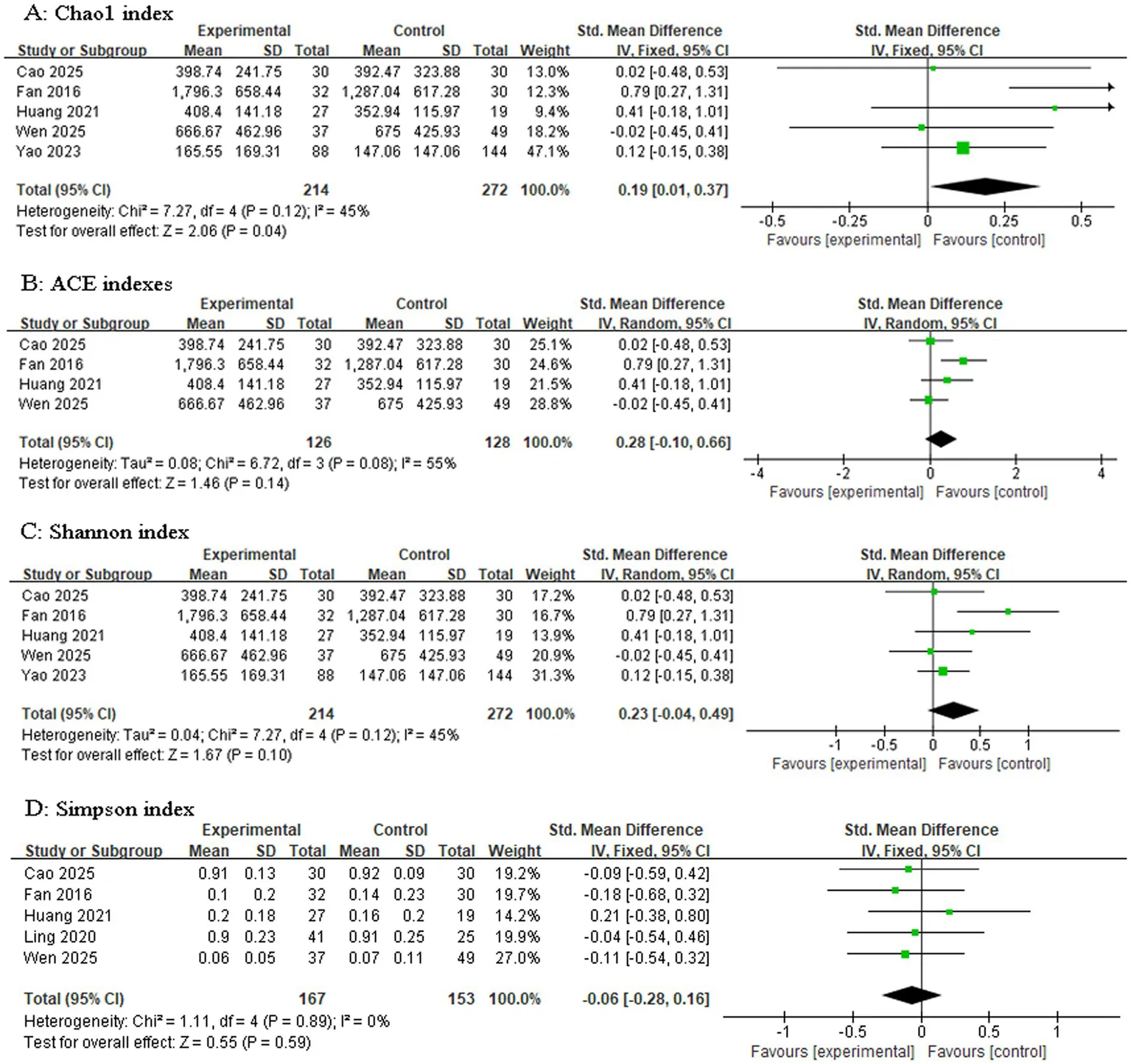
Forest plots showing comparisons of the Chao1 **(A)**, ACE **(B)**, Shannon **(C)**, and Simpson **(D)** indices between individuals with post-stroke mood disorders and healthy controls.

Six studies (Cao N. et al., 2025; Cao Y. et al., 2025; Fan et al., 2016; Huang et al., 2021; Ling et al., 2020a; Ling et al., 2020b; Wen et al., 2025; Yao et al., 2023) investigated the link between gut microbiota and post-stroke mood disorders. The Chao1 showed statistically significant differences between groups (95% CI: 0.19 [0.01, 0.37], *p* = 0.04, *I*^2^ = 45%) (Figure 3A). None reported significant between-group differences in α-diversity indices such as ACE, Simpson, or Shannon (Figures 3B–D).

#### 3.4.3 Gut microbiome α-diversity in post-stroke infection

Seven studies (Haak et al., 2021; He et al., 2024; Li et al., 2023; Lin et al., 2025; Luo et al., 2024; Yoshimura et al., 2025; Zheng et al., 2022) examined gut microbiota in stroke with infection. No significant differences were observed between groups across α-diversity indices, including ACE, Chao1, Simpson, Shannon, and observed species richness (Supplementary Figure S12).

### 3.5 Gut microbiome taxonomy in ischemic stroke

#### 3.5.1 Associations between gut microbial phyla and ischemic stroke

Ten studies (Chen et al., 2021; Fan et al., 2016; Gu et al., 2021; Li T. et al., 2022; Li Y. M. et al., 2022; Lledós et al., 2025; Pilon et al., 2025; Wang et al., 2018; Xiang et al., 2020; Xu et al., 2021; Yao et al., 2023) investigating gut microbiota at the phylum level in ischemic stroke patients showed no statistically significant differences in the abundance of *Actinobacteria*, *Bacteroidetes*, *Firmicutes*, *Fusobacteria*, *Proteobacteria*, *Synergistetes* and *Verrucomicrobia* (Supplementary Figure S13).

#### 3.5.2 Associations between gut microbial families and ischemic stroke

**Figure 4:**
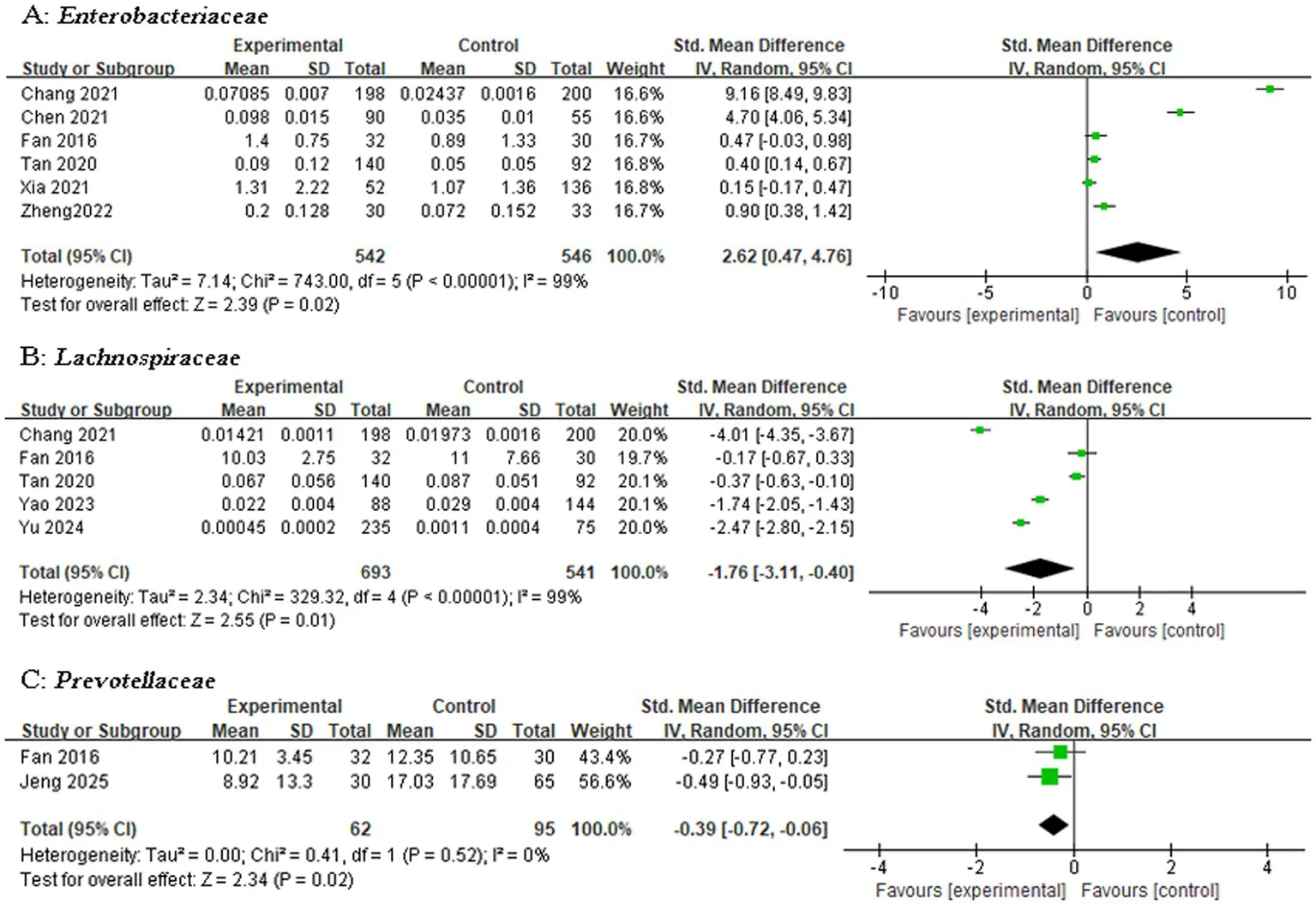
Forest plots illustrating family-level differences in gut microbiota between ischemic stroke patients and healthy controls. Panels show **(A)** *Enterobacteriaceae*, **(B)** *Lachnospiraceae*, and **(C)** *Prevotellaceae.*

A pooled analysis of nine studies (Chang et al., 2021; Chen et al., 2021; Fan et al., 2016; Jeng et al., 2025; Tan et al., 2021; Xia et al., 2021; Yao et al., 2023; Yu et al., 2024; Zheng et al., 2022) examining gut microbiota at the family level revealed a consistent association with ischemic stroke. Specifically, ischemic stroke patients showed elevated abundances of *Enterobacteriaceae*, and significantly reduced abundances of *Lachnospiraceae* and *Prevotellaceae* compared with controls, with mean differences (95% CI of 2.62 [0.47, 4.76], *p* = 0.02, *I*^2^ = 99%, −1.76 [−3.11, −0.40], *p* = 0.01, *I*^2^ = 99%, and −0.39 [−0.72, −0.06], *p* = 0.02, *I*^2^ = 0%), respectively (Figure 4). In contrast, no statistically significant differences were observed for *Butyricicoccaceae*, *Porphyromonadaceae*, or *Ruminococcaceae* (Supplementary Figure S14).

#### 3.5.3 Associations between gut microbial genera and ischemic stroke

**Figure 5:**
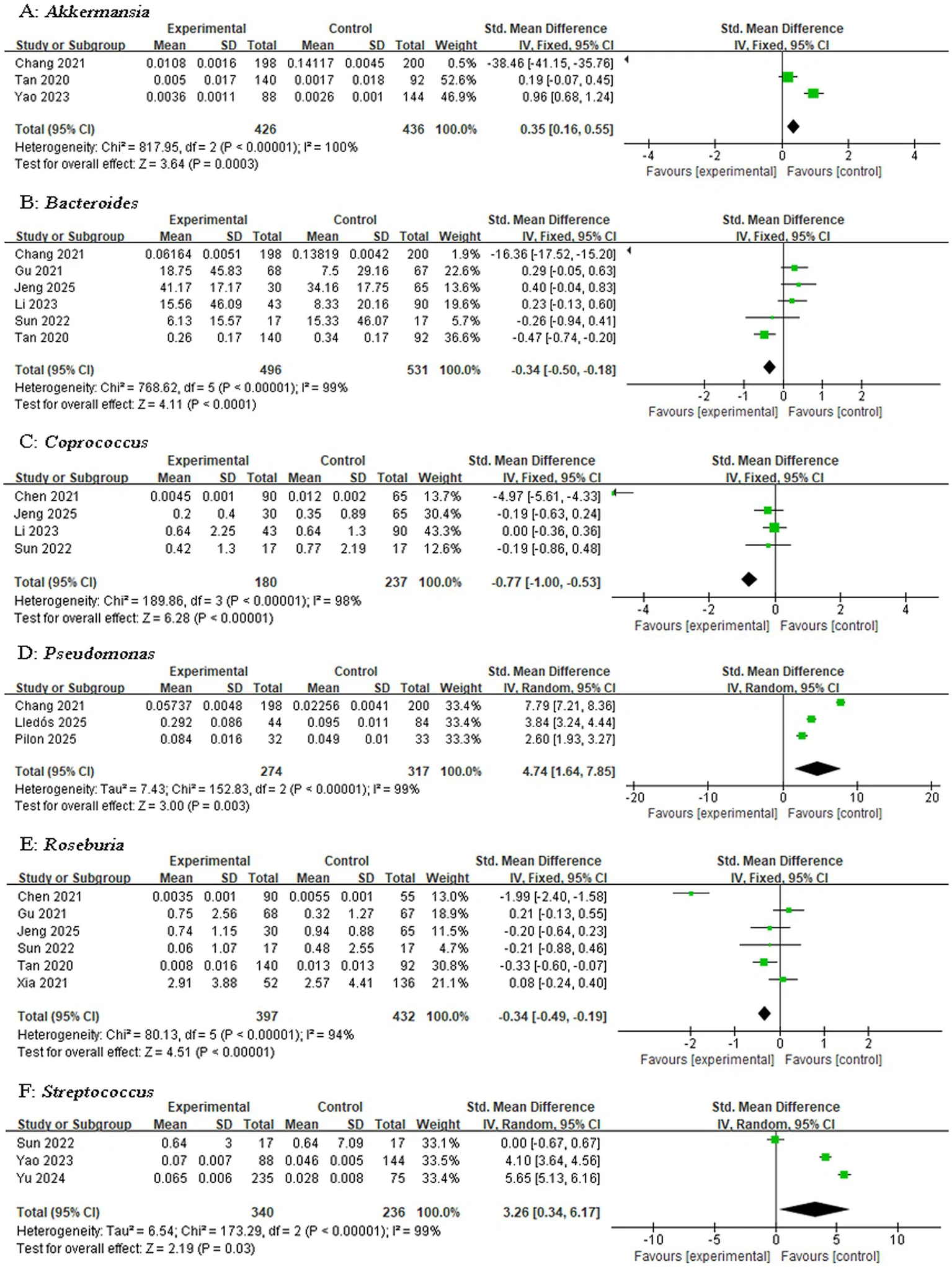
Forest plots showing genus-level differences in gut microbiota between patients with ischemic stroke and healthy controls. The genera presented are *Akkermansia* **(A)**, *Bacteroides* **(B)**, *Coprococcus* **(C)**, *Pseudomonas* **(D)**, *Roseburia* **(E)**, and *Streptococcus* **(F)**.

A total of 18 studies (Chang et al., 2021; Chen et al., 2021; Gu et al., 2021; Jeng et al., 2025; Kang et al., 2021; Li T. et al., 2022; Li Y. M. et al., 2022; Li et al., 2023; Lledós et al., 2025; Luo et al., 2024; Pilon et al., 2025; Sun et al., 2022; Tan et al., 2021; Xia et al., 2021; Xiang et al., 2020; Xu et al., 2021; Yao et al., 2023; Yu et al., 2024; Zheng et al., 2022) investigated the associations between gut microbiota at the genus level and ischemic stroke. Significant between-group differences were observed in the abundances of *Akkermansia*, *Bacteroides*, *Coprococcus*, *Pseudomonas*, *Roseburia*, and *Streptococcus* (95% CI: *Akkermansia*: 0.35 [0.16, 0.55], *p* = 0.0003, *I*^2^ = 100%; *Bacteroides*: −0.34 [−0.50, −0.18], *p* < 0.0001, *I*^2^ = 99%; *Coprococcus*: −0.77 [−1.00, −0.53], *p* < 0.00001, *I*^2^ = 98%; *Pseudomonas*: 4.74 [1.64, 7.85], *p* = 0.003, *I*^2^ = 99%; *Roseburia*: −0.34 [−0.49, −0.19], *p* < 0.00001, *I*^2^ = 94%; *Streptococcus*: 3.26 [0.34, 6.17], *p* = 0.03, *I*^2^ = 99%) (Figure 5). In contrast, no significant differences were found for *Bilophila*, *Blautia*, *Butyricicoccus*, *Enterococcus*, *Faecalibacterium*, *Lactobacillus*, or *Synergistota* between the two groups (Supplementary Figure S15).

### 3.6 Publication bias, sensitivity analyses, and meta-regression

Publication bias was not detected in most analyses, except for the overall ACE and Simpson indexes. For the ACE index, trim-and-fill imputation of two hypothetical studies resulted in an effect of 0.11 (95% CI: −0.081 to 0.302), with no reversal. In contrast, imputing 11 studies for the Simpson index produced an effect of −0.315 (95% CI: −0.496 to −0.135), indicating a clear reversal (Supplementary Table S3). Sensitivity analysis revealed that the observed gut microbiota alterations in ischemic stroke patients were not substantially influenced by any individual study (Supplementary Figure S16). Based on a random-effects meta-regression, none of the examined factors—year, 16S rRNA sequencing target region, literature quality scores, or study country—were found to be a major source of heterogeneity (Supplementary Table S4).

## 4 Discussion

Our systematic review and meta-analysis evaluated the connection between ischemic stroke and gut microbiota, elucidating key alterations in microbial diversity and taxonomy that offer a framework for subsequent studies. Although overall gut microbiota diversity was similar between stroke patients and controls, subgroup analysis showed changes in observed species among those with cognitive impairment, and changes in the Chao1 index among patient with mood disorders. Analysis of gut microbiota taxonomy in ischemic stroke patients revealed no significant phylum-level alterations, but family-level analysis identified increased *Enterobacteriaceae* and reduced *Lachnospiraceae* and *Prevotellaceae*, as well as differential abundances of six bacterial genera.

Assessment of gut microbiota includes α-diversity and β-diversity. Common α-diversity metrics—such as the Shannon and Simpson indices, as well as Chao1, ACE, and observed species richness—quantify both the species richness and evenness of the microbial community in a given sample (Willis, 2019). Our results showed that the two groups were statistically indistinguishable across multiple α-diversity metrics, including the Shannon index (species richness and evenness), the Chao1 and ACE indices (estimated richness), and the Simpson index (dominance). In agreement with previous studies (Bao et al., 2024), our results confirm that α-diversity levels were comparable between ischemic stroke patients and healthy controls, with no statistically significant difference observed. Similarly, Pilon et al. observed comparable α-diversity levels in patients with severe versus moderate stroke (Pilon et al., 2025). However, α-diversity analysis revealed reduced bacterial richness in the microbiota of patients with unfavorable versus favorable outcomes (Lledós et al., 2025). We analyzed data from 59 studies comprising 7,463 patients-the largest cohort to date-which included recently published trials with improved design and assessment. Our findings demonstrate that altered microbial α-diversity is not a common characteristic in ischemic stroke patients. However, post-stroke gut microbiota changes are time-dependent and may not be fully captured in cross-sectional studies. Additionally, comorbidities such as hypertension and diabetes may act as confounders, increasing within-group heterogeneity and reducing the detectability of statistical differences in stroke patients. Therefore, the lack of significant differences in α-diversity should not be interpreted as evidence of an undisturbed gut microbiota. Ischemic stroke may not directly correlate with overall changes in gut microbiota diversity, it could be linked to shifts in the composition of specific functional groups.

Limited evidence exists regarding stroke subtype-specific gut microbiota alterations. This study further examines these alterations in stroke patients with comorbid cognitive, mood, and inflammatory disorders. The cognitive impairment and mood disorder subgroups require special attention. Among stroke patients with cognitive impairment, changes in microbial observed species richness were detected, while those with affective disorders showed altered Chao1 indices. These findings may link microbial diversity to cognitive decline and emotional disturbance. This is consistent with earlier meta-analyses comparing post-stroke cognitive impairment and affective disorders to healthy controls. Recent studies point to alterations in the structure and abundance of gut microbiota in individuals with vascular cognitive impairment (Olejnik and Golenia, 2024). Reduced levels of microbial-derived short-chain fatty acids (SCFAs), stemming from gut microbiota dysbiosis, have been linked to the emergence of post-stroke cognitive impairment (Liu et al., 2020). Post-stroke depression patients may exhibit marked shifts in species composition and microbial community structure (Chen et al., 2025). These results suggest that different microbial metrics may underlie distinct neuropsychiatric sequelae. These observations align with the emerging concept of the gut–brain axis and highlight the potential for microbiome-based stratification in stroke rehabilitation. However, these findings, derived from an exploratory analysis with a small sample size, heterogeneity, and uncontrolled confounders, should be interpreted as hypothesis-generating rather than conclusive. They warrant further validation in future confirmatory studies.

For the first time, this meta-analysis revealed that stroke-associated gut microbiota dysbiosis is specifically observed at the family level. In our study, dysbiosis was marked by an increased abundance of pro-inflammatory *Enterobacteriaceae* and a reduced abundance of anti-inflammatory *Lactobacillaceae*. This shift occurred not at the phylum level, but rather between functionally more defined taxa at the family level. *Enterobacteriaceae*—a family of opportunistic pathogens typified by *Escherichia coli*—may drive systemic lipopolysaccharide accumulation (Liu et al., 2026), incite inflammatory cascades, aggravate ischemic brain damage, and compromise neurological recovery after stroke (Xu et al., 2021). Conversely, reduced *Lachnospiraceae* abundance may lower intestinal butyrate output, potentially weakening neuroprotection and compromising blood–brain barrier function after brain injury (Liu et al., 2026). Our findings suggest that these two bacterial families may contribute to the pathogenesis of stroke, thereby supporting gut microbiota modulation and butyrate supplementation as promising therapeutic directions.

At the genus level, we observed significant changes in *Akkermansia*, *Bacteroides*, *Pseudomonas*, *Coprococcus*, *Roseburia*, and *Streptococcus*. *Akkermansia muciniphila* is crucial for maintaining intestinal mucus integrity, and its loss disrupts the barrier, promoting bacterial translocation and exacerbating post-stroke inflammation (Bao et al., 2022). Conversely, *Pseudomonas*, as an opportunistic pathogen, contains lipopolysaccharide that enters bloodstream via damaged intestinal barriers, inducing systemic inflammation that exacerbates ischemia–reperfusion injury (Xia et al., 2025). The gut microbiota breaks down dietary fiber, yielding SCFAs, chiefly acetate, propionate, and butyrate, which appear to protect against ischemic stroke (Fang et al., 2023). Notably, excessive intestinal *Streptococcus* may consume SCFAs precursors and generate harmful metabolites, whereas *Bacteroides* may produce SCFAs (Peh et al., 2022; Yao et al., 2025). *Coprococcus* and *Roseburia*, both key butyrate producers within *Lachnospiraceae*, exert anti-inflammatory effects through histone deacetylase inhibition, microglial quiescence, and improvements in sensorimotor and reflex function (Fang et al., 2023; Snodgrass and Velayudhan, 2026). Beyond SCFAs deficiency, the altered gut microbiota in stroke patients—characterized by altered abundances of *Bacteroides*, *Roseburia*, and *Streptococcus*, bacteria involved in trimethylamine production—may raise circulating trimethylamine-N-oxide levels, thereby worsening neuroinflammation and brain injury (Peh et al., 2022). Collectively, these genus-level changes suggest that stroke patients may develop a distinct gut dysbiosis pattern, marked by an increase in pro-inflammatory and barrier-disrupting bacteria, along with a reduction in taxa that support barrier integrity and immune balance. This shift reflects a selective functional decline—particularly in barrier maintenance and SCFAs production—alongside the rise of opportunistic pathogens. Unlike general microbial depletion, this signature points to mechanistically relevant targets in stroke pathophysiology.

### 4.1 Heterogeneity and publication bias

Our findings revealed substantial heterogeneity across studies. Despite extensive subgroup analyses, meta-regression, and sensitivity analyses, we could not identify a single factor fully explaining the heterogeneity. This variability likely stems from complex interactions among multiple unstandardized factors in the original studies, including host genetics, dietary habits, sampling sites and time points, and importantly inconsistent reporting of stroke phase, severity, and key confounders. Most primary studies did not report these variables, preventing further stratified analyses or covariate adjustment. Therefore, this meta-analysis examined whether gut microbiota changes are a consistent feature across the stroke continuum, not just in one phase or complication. The pooled estimates are correlational, not causal, and should be viewed as exploratory trends rather than precise quantitative summaries. Sensitivity analyses excluding antibiotic/probiotic users and subgroup analyses by geographic region yielded consistent results, indirectly supporting robustness, though not fully addressing these concerns.

The primary studies in this meta-analysis used heterogeneous methods, including various sequencing platforms, different 16S rRNA target regions (ranging from V3 to V4–V5), a mix of metagenomic and 16S amplicon approaches, and inconsistent bioinformatic pipelines. Such technical variation is known to significantly affect taxonomic classification and abundance estimates, introducing potential bias. To partially address this, we conducted subgroup analyses stratified by sequencing region where feasible. The consistent results across these analyses offer some reassurance. Nevertheless, this methodological heterogeneity remains a limitation that should be considered when interpreting our pooled estimates.

Our primary meta-analysis found no significant difference in overall α-diversity between groups. Publication bias was suggested for the ACE and Simpson indices. After trim-and-fill adjustment, the ACE index remained non-significant, while the Simpson index became significant—but this result requires caution due to discordant Begg’s and Egger’s tests and the assumptions of the trim-and-fill method. Sensitivity analyses confirmed no single study drove the overall estimates. Overall, the current evidence, especially for the Simpson index, may be influenced by publication bias. Therefore, these results should be interpreted cautiously. Future high-quality, large-sample randomized controlled trials are needed to confirm this finding.

### 4.2 Strengths and weaknesses

Our study integrates α-diversity, taxonomic shifts, and comorbidity profiles into a unified framework that connects ecological patterns with clinically actionable microbiota signatures. Our findings link microbial alterations to actionable pathological mechanisms including cognitive decline, impaired SCFAs metabolism, and opportunistic pathogen expansion, identifying specific therapeutic targets for probiotic or metabolite-based interventions. Restoring *A. muciniphila* and butyrate producers through prebiotics, probiotics, or bioactive metabolites holds promise as an adjunctive strategy for stroke recovery, while curbing *Enterobacteriaceae* expansion may dampen systemic inflammation and secondary brain injury—hypotheses that await validation in randomized trials.

However, our findings should be evaluated in light of several limitations. First, α-diversity analyses were underpowered due to the scarcity of studies reporting composite measures; the predominant focus on individual indices (e.g., Shannon, Chao1) sacrificed statistical robustness for metric-specific granularity. β-Diversity is usually represented as distance matrices (e.g., Bray-Curtis, Jaccard, UniFrac) rather than single values. Hence, we did not perform statistical pooling of β-diversity. Second, the timing of fecal sample collection varied considerably among the included studies. Gut microbiota composition is known to change dynamically after stroke, driven by factors such as systemic inflammation, antibiotic use, and dietary changes during recovery. Pooling samples from different disease stages in cross-sectional designs may obscure stage-specific microbial patterns and contribute to the marked heterogeneity observed in our analyses. As a result, it is not possible to determine temporal relationships or causality. Whether the observed microbial changes precede stroke (as risk factors), result from acute stroke, or reflect long-term post-stroke adaptations remains unclear. Future studies should adopt standardized, longitudinal sampling protocols to better capture the temporal dynamics of the gut microbiome and its link to stroke outcomes. Third, our analysis was constrained by incomplete data on important clinical confounders in the original studies, including antibiotic use, proton pump inhibitor use, enteral feeding, intensive care unit admission, and nutritional status. Most included studies did not consistently report these variables, preventing us from performing subgroup analyses or adjusting for their effects. Our pooled findings should therefore be interpreted as correlational patterns indicating overall dysbiosis in the stroke continuum, not as precise causal estimates. Fourth, this study did not examine gut microbiota metabolites; thus, future work should determine their effects on ischemic stroke. Fifth, many included studies had relatively low NOS scores. To assess the potential impact on our findings, we performed a sensitivity analysis excluding low-quality studies and a subgroup analysis comparing low- and high-quality studies. The pooled effect remained stable in both direction and statistical significance in the sensitivity analysis, and the subgroup analysis showed no significant difference between groups. Nonetheless, the presence of low-quality evidence should be considered when interpreting the results, and caution is warranted when applying the findings to clinical practice. Sixth, the application of diverse screening tools (e.g., MMSE vs. MoCA) across the included primary studies may have introduced both clinical heterogeneity and measurement bias. Therefore, future prospective investigations should utilize standardized and harmonized neuropsychological assessments to validate our findings. Seventh, as most of the included original studies were conducted in Chinese populations, it remains unclear whether our conclusions can be generalized to other ethnic or regional groups. Large-scale, multi-center cohort studies covering diverse regions and ethnicities are therefore needed to validate the applicability of our findings. Eighth, many significant taxonomic differences we found had very small effect sizes. Although minor shifts in key taxa might still affect microbial communities, their functional and clinical relevance is unclear. Thus, our results are hypothesis-generating, and future work should test whether such modest taxonomic changes are clinically meaningful. Finally, this study did not investigate gut microbiota at class, order, and species levels, preventing a full understanding of microbiota. Thus, further research is warranted to clarify this.

Future research should prioritize several directions. First, large-scale prospective cohort studies with repeated microbiome sampling and standardized assessment protocols are urgently needed to establish temporal relationships and clarify whether post-stroke microbial changes precede or simply reflect functional decline. Second, since most existing studies have used 16S rRNA amplicon sequencing, future work should adopt shotgun metagenomics to characterize functional gene profiles and strain-level differences. Third, future cohort studies should combine metagenomics with metabolomics (e.g., untargeted profiling of serum or fecal metabolites) to pinpoint specific bioactive molecules—such as SCFAs, secondary bile acids, or tryptophan derivatives—that mediate host–microbiome interactions. Fourth, beyond observational data, mechanistic experiments using germ-free models, fecal microbiota transplantation, and defined bacterial consortia are needed to establish causal links and identify candidate therapeutic strains or metabolites. Finally, well-powered randomized controlled trials are needed to validate the safety and efficacy of microbiota-based interventions in stroke patients, while Mendelian randomization could further strengthen causal inference by leveraging host genetic variants as instrumental variables for microbial traits.

## 5 Conclusion

Ischemic stroke is characterized by taxonomic gut dysbiosis, whereas reduced α-diversity occurs restricted to patients with post-stroke cognitive impairment and mood disorders. Future studies that integrate multi-omics data and large-scale cohort designs are needed to establish causal relationships between gut microbiota and ischemic stroke.

## Data Availability

The original contributions presented in the study are included in the article/Supplementary material, further inquiries can be directed to the corresponding authors.
