# Supplementary material for: The gut microbiota dysbiosis in ischemic stroke: a systematic review and meta-analysis of microbial diversity and taxonomic alterations
Source: Front Aging Neurosci. 2026 Aug 31;18:1914614. doi: 10.3389/fnagi.2026.1914614 (PMC13572240; doi:10.3389/fnagi.2026.1914614)
Supplement: Supplementary file 1 [file Data_sheet__1.docx]

Supplementary FIGURE S1

Depicts the cross‑sectional association between the Shannon index of the gut microbiome and ischemic stroke.

Supplementary FIGURE S2

Subgroup analysis of the cross-sectional association between gut microbiome Shannon index and ischemic stroke, stratified by 16S rRNA sequencing target region.

Supplementary FIGURE S3

Subgroup analysis of cross-sectional gut microbiome Shannon index–ischemic stroke association, stratified by study quality scores.

Supplementary FIGURE S4

Subgroup analysis of cross-sectional gut microbiome Shannon index–ischemic stroke association, stratified by region (Asia vs. non-Asia).

Supplementary FIGURE S5

Depicts the cross‑sectional relationship of gut microbiome Chao1 index with ischemic stroke.

Supplementary FIGURE S6

Depicts the cross‑sectional association between the gut microbiome ACE indexes and ischemic stroke.

Supplementary FIGURE S7

Depicts the cross‑sectional association between the gut microbiome Simpson index and ischemic stroke.

Supplementary FIGURE S8

Subgroup analysis of the cross-sectional association between gut microbiome Simpson index and ischemic stroke, stratified by 16S rRNA sequencing target region.

Supplementary FIGURE S9

Subgroup analysis of cross-sectional gut microbiome Simpson index–ischemic stroke association, stratified by study quality scores.

Supplementary FIGURE S10

Subgroup analysis of cross-sectional gut microbiome Simpson index–ischemic stroke association, stratified by region (Asia vs. non-Asia).

Supplementary FIGURE S11

Depicts the cross‑sectional association between the gut microbiome observed species richness and ischemic stroke.

Supplementary FIGURE S12

Forest plots illustrating comparisons of the ACE indexes (A), Chao1 index (B), Simpson index (C), Shannon index (D), and observed species richness (E) between post-stroke infection patients and healthy controls.

Supplementary FIGURE S13

Forest plots comparing phylum-level gut microbiota alterations between ischemic stroke patients and healthy controls. Panels represent: (A) *Actinobacteria*, (B) *Bacteroidetes*, (C) *Firmicutes*, (D) *Fusobacteria*, (E) *Proteobacteria*, (F) *Synergistetes*, (G) *Verrucomicrobia*.

Supplementary FIGURE S14

Forest plots comparing family-level gut microbiota alterations between ischemic stroke patients and healthy controls. Panels represent: (A) *Butyricicoccaceae*, (B) *Porphyromonadaceae*, (C) *Ruminococcaceae*.

Supplementary FIGURE S15

Forest plots of alterations of gut microbiota at the genus level between ischemic stroke and healthy controls spectrum, including (A) *Bilophila*, (B) *Blautia*, (C) *Butyricicoccus*, (D) *Enterococcus*, (E) *Faecalibacterium*, (F) *Lactobacillus*, (G) *Synergistota*.

Supplementary FIGURE S16

Sensitivity analysis of the included literature.
