# Supplementary material for: The gut microbiota dysbiosis in ischemic stroke: a systematic review and meta-analysis of microbial diversity and taxonomic alterations
Source: Front Aging Neurosci. 2026 Aug 31;18:1914614. doi: 10.3389/fnagi.2026.1914614 (PMC13572240; doi:10.3389/fnagi.2026.1914614)
Supplement: Supplementary file 2 [file Data_sheet__2.doc]

**Supplementary Table 1.** The search strategy

Database: PubMed

Date of Search: October 07, 2025

Search Strategy:

--------------------------------------------------------------------------------

("stroke"[MeSH] OR "stroke"[tiab] OR strokes[tiab] OR "Cerebrovascular Accident"[tiab] OR "Cerebrovascular Accidents"[tiab] OR "ischemic stroke"[tiab] OR "Acute ischemic stroke"[tiab] OR "ischaemic stroke"[tiab] OR "CVA (Cerebrovascular Accident)"[tiab] OR "CVAs (Cerebrovascular Accident)"[tiab] OR "Cerebrovascular Apoplexy"[tiab] OR "Apoplexy, Cerebrovascular"[tiab] OR "Vascular Accident, Brain"[tiab] OR "Brain Vascular Accident"[tiab] OR "Brain Vascular Accidents"[tiab] OR "Vascular Accidents, Brain"[tiab] OR "Cerebrovascular Stroke"[tiab] OR "Cerebrovascular Strokes"[tiab] OR "Stroke, Cerebrovascular"[tiab] OR "Strokes, Cerebrovascular"[tiab] OR Apoplexy[tiab] OR "Cerebral Stroke"[tiab] OR "Cerebral Strokes"[tiab] OR "Stroke, Cerebral"[tiab] OR "Strokes, Cerebral"[tiab] OR "Stroke, Acute"[tiab] OR "Acute Stroke"[tiab] OR "Acute Strokes"[tiab] OR "Strokes, Acute"[tiab] OR "Cerebrovascular Accident, Acute"[tiab] OR "Acute Cerebrovascular Accident"[tiab] OR "Acute Cerebrovascular Accidents"[tiab] OR "Cerebrovascular Accidents, Acute"[tiab] OR "stroke recovery"[tiab] OR "stroke unit"[tiab] OR thrombolysis[tiab] OR "acute cerebral infarction"[tiab] OR "cerebral infarction"[tiab] OR "delayed cerebral ischemia"[tiab] OR infarction[tiab])

AND

("Gastrointestinal Microbiome"[MeSH] OR "Gastrointestinal Microbiome"[tiab] OR "Gastrointestinal Microbiomes"[tiab] OR "Microbiome, Gastrointestinal"[tiab] OR "Gut Microbiome"[tiab] OR "Gut Microbiomes"[tiab] OR "Microbiome, Gut"[tiab] OR "Gut Microflora"[tiab] OR "Microflora, Gut"[tiab] OR "Gut Microbiota"[tiab] OR "Gut Microbiotas"[tiab] OR "gut dysbiosis"[tiab] OR "gut-brain axis"[tiab] OR microbiota[tiab] OR "microbiota-gut-brain axis"[tiab] OR probiotics[tiab] OR "Microbiota, Gut"[tiab] OR "Gastrointestinal Flora"[tiab] OR "Flora, Gastrointestinal"[tiab] OR "Gut Flora"[tiab] OR "Flora, Gut"[tiab] OR "Gastrointestinal Microbiota"[tiab] OR "Gastrointestinal Microbiotas"[tiab] OR "Microbiota, Gastrointestinal"[tiab] OR "Gastrointestinal Microbial Community"[tiab] OR "Gastrointestinal Microbial Communities"[tiab] OR "Microbial Community, Gastrointestinal"[tiab] OR "Gastrointestinal Microflora"[tiab] OR "Microflora, Gastrointestinal"[tiab] OR "Gastric Microbiome"[tiab] OR "Gastric Microbiomes"[tiab] OR "Microbiome, Gastric"[tiab] OR "Intestinal Microbiome"[tiab] OR "Intestinal Microbiomes"[tiab] OR "Microbiome, Intestinal"[tiab] OR "Intestinal Microbiotas"[tiab] OR "Microbiota, Intestinal"[tiab] OR "Intestinal Microflora"[tiab] OR "Microflora, Intestinal"[tiab] OR "Intestinal Flora"[tiab] OR "Flora, Intestinal"[tiab] OR "Enteric Bacteria"[tiab] OR "Bacteria, Enteric"[tiab] OR "Intestinal Microbiota"[tiab])

Database: Cochrane Database of Systematic Reviews (CDSR) & Cochrane Central Register of Controlled Trials (CENTRAL)

Date of Search: October 07, 2025

Search Strategy:

--------------------------------------------------------------------------------

("stroke":ti,ab,kw OR strokes:ti,ab,kw OR "Cerebrovascular Accident":ti,ab,kw OR "Cerebrovascular Accidents":ti,ab,kw OR "ischemic stroke":ti,ab,kw OR "Acute ischemic stroke":ti,ab,kw OR "ischaemic stroke":ti,ab,kw OR "CVA (Cerebrovascular Accident)":ti,ab,kw OR "CVAs (Cerebrovascular Accident)":ti,ab,kw OR "Cerebrovascular Apoplexy":ti,ab,kw OR "Apoplexy, Cerebrovascular":ti,ab,kw OR "Vascular Accident, Brain":ti,ab,kw OR "Brain Vascular Accident":ti,ab,kw OR "Brain Vascular Accidents":ti,ab,kw OR "Vascular Accidents, Brain":ti,ab,kw OR "Cerebrovascular Stroke":ti,ab,kw OR "Cerebrovascular Strokes":ti,ab,kw OR "Stroke, Cerebrovascular":ti,ab,kw OR "Strokes, Cerebrovascular":ti,ab,kw OR Apoplexy:ti,ab,kw OR "Cerebral Stroke":ti,ab,kw OR "Cerebral Strokes":ti,ab,kw OR "Stroke, Cerebral":ti,ab,kw OR "Strokes, Cerebral":ti,ab,kw OR "Stroke, Acute":ti,ab,kw OR "Acute Stroke":ti,ab,kw OR "Acute Strokes":ti,ab,kw OR "Strokes, Acute":ti,ab,kw OR "Cerebrovascular Accident, Acute":ti,ab,kw OR "Acute Cerebrovascular Accident":ti,ab,kw OR "Acute Cerebrovascular Accidents":ti,ab,kw OR "Cerebrovascular Accidents, Acute":ti,ab,kw OR "stroke recovery":ti,ab,kw OR "stroke unit":ti,ab,kw OR thrombolysis:ti,ab,kw OR "acute cerebral infarction":ti,ab,kw OR "cerebral infarction":ti,ab,kw OR "delayed cerebral ischemia":ti,ab,kw OR infarction:ti,ab,kw)

AND

("Gastrointestinal Microbiome":ti,ab,kw OR "Gastrointestinal Microbiomes":ti,ab,kw OR "Microbiome, Gastrointestinal":ti,ab,kw OR "Gut Microbiome":ti,ab,kw OR "Gut Microbiomes":ti,ab,kw OR "Microbiome, Gut":ti,ab,kw OR "Gut Microflora":ti,ab,kw OR "Microflora, Gut":ti,ab,kw OR "Gut Microbiota":ti,ab,kw OR "Gut Microbiotas":ti,ab,kw OR "gut dysbiosis":ti,ab,kw OR "gut-brain axis":ti,ab,kw OR microbiota:ti,ab,kw OR "microbiota-gut-brain axis":ti,ab,kw OR probiotics:ti,ab,kw OR "Microbiota, Gut":ti,ab,kw OR "Gastrointestinal Flora":ti,ab,kw OR "Flora, Gastrointestinal":ti,ab,kw OR "Gut Flora":ti,ab,kw OR "Flora, Gut":ti,ab,kw OR "Gastrointestinal Microbiota":ti,ab,kw OR "Gastrointestinal Microbiotas":ti,ab,kw OR "Microbiota, Gastrointestinal":ti,ab,kw OR "Gastrointestinal Microbial Community":ti,ab,kw OR "Gastrointestinal Microbial Communities":ti,ab,kw OR "Microbial Community, Gastrointestinal":ti,ab,kw OR "Gastrointestinal Microflora":ti,ab,kw OR "Microflora, Gastrointestinal":ti,ab,kw OR "Gastric Microbiome":ti,ab,kw OR "Gastric Microbiomes":ti,ab,kw OR "Microbiome, Gastric":ti,ab,kw OR "Intestinal Microbiome":ti,ab,kw OR "Intestinal Microbiomes":ti,ab,kw OR "Microbiome, Intestinal":ti,ab,kw OR "Intestinal Microbiotas":ti,ab,kw OR "Microbiota, Intestinal":ti,ab,kw OR "Intestinal Microflora":ti,ab,kw OR "Microflora, Intestinal":ti,ab,kw OR "Intestinal Flora":ti,ab,kw OR "Flora, Intestinal":ti,ab,kw OR "Enteric Bacteria":ti,ab,kw OR "Bacteria, Enteric":ti,ab,kw OR "Intestinal Microbiota":ti,ab,kw)

Database: Embase

Date of Search: October 07, 2025

Search Strategy:

--------------------------------------------------------------------------------

(stroke/exp OR cerebrovascular accident/exp OR cerebral infarction/exp OR ischemic stroke/exp OR acute ischemic stroke/exp OR apoplexy/exp OR cerebral stroke/exp OR stroke/de OR cerebrovascular accident/de OR cerebral infarction/de OR ischemic stroke/de OR acute ischemic stroke/de OR apoplexy/de OR cerebral stroke/de OR (stroke* OR "cerebrovascular accident*" OR "cerebrovascular accidents" OR "ischemic stroke*" OR "acute ischemic stroke*" OR "ischaemic stroke*" OR CVA OR CVAs OR "cerebrovascular apoplexy" OR "apoplexy, cerebrovascular" OR "vascular accident, brain" OR "brain vascular accident*" OR "cerebrovascular stroke*" OR apoplexy OR "cerebral stroke*" OR "acute stroke*" OR "stroke recovery" OR "stroke unit" OR thrombolysis OR "acute cerebral infarction" OR "cerebral infarction" OR "delayed cerebral ischemia" OR infarction))

AND

(gastrointestinal microbiome/exp OR gut microbiome/exp OR gut microbiota/exp OR gut flora/exp OR enteric bacteria/exp OR intestinal microbiome/exp OR intestinal microbiota/exp OR intestinal flora/exp OR gastric microbiome/exp OR microbiota/exp OR gut-brain axis/exp OR microbiota-gut-brain axis/exp OR probiotics/exp OR gastrointestinal flora/exp OR gastrointestinal microbiota/exp OR gastrointestinal microbial community/exp OR (("gastrointestinal microbiome*" OR "gastrointestinal microbiomes" OR "microbiome, gastrointestinal" OR "gut microbiome*" OR "gut microbiomes" OR "microbiome, gut" OR "gut microflora" OR "microflora, gut" OR "gut microbiota*" OR "gut microbiotas" OR "gut dysbiosis" OR "gut-brain axis" OR microbiota OR "microbiota-gut-brain axis" OR probiotics OR "microbiota, gut" OR "gastrointestinal flora" OR "flora, gastrointestinal" OR "gut flora" OR "flora, gut" OR "gastrointestinal microbiota*" OR "gastrointestinal microbiotas" OR "microbiota, gastrointestinal" OR "gastrointestinal microbial community*" OR "gastrointestinal microbial communities" OR "microbial community, gastrointestinal" OR "gastrointestinal microflora" OR "microflora, gastrointestinal" OR "gastric microbiome*" OR "gastric microbiomes" OR "microbiome, gastric" OR "intestinal microbiome*" OR "intestinal microbiomes" OR "microbiome, intestinal" OR "intestinal microbiotas" OR "microbiota, intestinal" OR "intestinal microflora" OR "microflora, intestinal" OR "intestinal flora" OR "flora, intestinal" OR "enteric bacteria" OR "bacteria, enteric" OR "intestinal microbiota")) OR gut dysbiosis/exp))

***************************
